# Impact of storage time in dried blood samples (DBS) and dried plasma samples (DPS) for point-of-care hepatitis C virus (HCV) RNA quantification and HCV core antigen detection

**DOI:** 10.1128/spectrum.01748-23

**Published:** 2023-09-01

**Authors:** Paloma Troyano-Hernáez, Pedro Herrador, Francisco Gea, Beatriz Romero-Hernández, Gabriel Reina, Agustín Albillos, Juan Carlos Galán, África Holguín

**Affiliations:** 1 Microbiology Department, HIV-1 Molecular Epidemiology Laboratory, Ramón y Cajal University Hospital-Ramón y Cajal Institute for Health Research (IRYCIS) and RITIP-CoRISpe, Madrid, Spain; 2 Microbiology Department, Ramón y Cajal University Hospital-Ramón y Cajal Institute for Health Research (IRYCIS), Madrid, Spain; 3 Gastroenterology Department, Ramón y Cajal University Hospital-Ramón y Cajal Institute for Health Research (IRYCIS), Madrid, Spain; 4 Biomedical Research Center in Epidemiology and Public Health (CIBERESP), Madrid, Spain; 5 Microbiology Department, Clínica Universidad de Navarra, Pamplona, Spain; 6 ISTUN, Institute of Tropical Health, Universidad de Navarra, Pamplona, Spain; 7 IdiSNA, Navarra Institute for Health Research, Pamplona, Spain; 8 Biomedical Research Center on Liver and Digestive Diseases (CIBEREHD), Madrid, Spain; 9 University of Alcalá, Madrid, Spain; Johns Hopkins Medicine, Baltimore, Maryland, USA

**Keywords:** HCV, DBS, DPS, POC, core antigen, dried blood, hepatitis C, viral load

## Abstract

The scale-up of hepatitis C virus (HCV) diagnosis and treatment requires affordable and simple tools to improve access to care, especially in low- and middle-income settings with limited infrastructure or high-risk populations. Dried blood and plasma samples (DBS and DPS) are useful alternative for hepatitis C detection in settings lacking adequate infrastructure. We evaluated the performance of DBS and DPS vs plasma in a point-of-care HCV RNA quantitative assay (Xpert HCV Viral Load-Cepheid), and compared HCV core antigen (HCVcAg) detection by the Architect HCV core antigen assay (Abbott) in DBS vs serum. The dried samples were stored at room temperature for different storage times to reproduce the time from sampling to testing in settings with centralized diagnosis or when testing mobile populations. HCV RNA quantification in DBS and DPS presented 100% sensitivity and specificity and a high correlation for up to 3 months of storage. HCV viremia showed a mean decrease of 0.5 log_10_ IU/mL (DBS) and 0.3 log_10_ IU/mL (DPS) for storage times up to 1 month. Architect HCVcAg detection presented high sensitivity/specificity (96%/100%) in DBS tested immediately after sampling, decreasing to 86% sensitivity after 7 days of storage. However, sensitivity increased when an optimized cut-off was applied for each storage time. We conclude that DBS and DPS are suitable samples for HCV RNA detection and quantification, being DPS more reliable for shorter storage times. DBS can be also used for HCVcAg qualitative detection and the sensitivity can be increased when adjusting the cut-off values.

**IMPORTANCE:**

Hepatitis C infection remains a global burden despite the effectiveness of antivirals. In the WHO roadmap to accomplish HCV elimination by 2030, HCV diagnosis is one of the main targets. However, identifying patients in resource-limited settings and high-risk populations with limited access to healthcare remains a challenge and requires innovative approaches that allow decentralized testing. The significance of our research is in verifying the good performance of dried samples for HCV diagnosis using two different diagnostics assays and considering the effect of room temperature storage in this sample format. We confirmed dried samples are an interesting alternative for HCV screening and reflex testing in resource-limited settings or high-risk populations.

## INTRODUCTION

Hepatitis C virus (HCV) can cause acute or chronic infection, posing a major global public health problem. Around 30% of infected persons spontaneously clear the virus within 6 months of infection without treatment, and the remaining 70% will develop chronic HCV infection with a risk of cirrhosis and hepatocellular carcinoma ranging from 15% to 30% within 20 years (1, 2). According to the World Health Organization (WHO), an estimated 58 million people globally have chronic HCV infection, with about 1.5 million new infections occurring every year but only 20% are diagnosed and 13% have been treated (1). In 2019, the WHO estimated 290,000 people died from hepatitis C, mostly from cirrhosis and carcinoma (1). The WHO has promoted viral hepatitis elimination as a public health problem by 2030, aiming for ≥90% of people with HCV diagnosed by 2030 (3).

Despite the widespread availability of curative treatment with direct-acting antivirals (4), a significant proportion of people with HCV remain undiagnosed and untreated, especially in certain population groups with high HCV prevalence such as HIV-coinfected men who have sex with men, people who inject drugs, hemodialysis patients, and immigrants (5, 6). Areas with high rates of infection are located in the Eastern Mediterranean Region and the European Region, where unsafe healthcare injections and injection drug use accounts for a substantial proportion of new infections, respectively (7). In Spain, where this study was performed, HCV seroprevalence between 2017 and 2018 was 0.85% (0.22% with active infection), increasing to 1.8% in some cities, as in Madrid where this study was conducted (7, 8).

Traditional microbiological HCV diagnosis consisted of a first step of serological techniques, based on the detection of anti-HCV antibodies (Abs-HCV) by enzyme immunoassays (EIA) or chemiluminescent immunoassays (CLIA), indicating current or past infection (2). A second step involved direct techniques of viral detection, such as RNA or HCV core antigen (HCVcAg) detection to identify viremic patients (2, 9). Several studies have validated the use of HCVcAg for the diagnosis of active infection compared to viral load (VL) determination, obtaining excellent sensitivity and specificity results (10 – 12). The diagnosis can be further simplified by reflex testing (one-step strategy) as recommended by the EASL (13) and the AASLD (14), which involves using the same sample to determine Ab-HCV and HCV RNA/HCVcAg (15).

However, eliminating HCV in some settings and population groups will require innovative approaches for sample collection that can facilitate the diagnosis, allowing to decentralize testing and treatment in lower-level health facilities (5, 16). Dried blood samples (DBS) are useful alternative for the diagnosis and monitoring of a wide variety of infectious diseases (17 – 19), including hepatitis C (20 – 23), in settings lacking adequate infrastructure (24) and in certain groups with limited access to medical care since they are easy to collect, store, and transport (20, 25). DBS preparation does not require specialized laboratories, cold chain, centrifuges, or other complex equipment at the sampling site. They can be prepared with a few drops of blood obtained in a minimally invasive manner, with a simple finger or heel prick, facilitating sampling when venipuncture is complicated or the availability of blood volume is limited. Furthermore, DBS can be drawn by minimally trained personnel and even self-collected by the patient (26). DBS present greater stability at room temperature (RT) (1–2 weeks) than whole blood (6 hours) or serum (24 hours), remaining stable for several years at low temperatures (17, 27). DBS room temperature storage effect on diagnosis has been more studied in other pathogens (28 – 31) than in HCV (23, 32, 33) and present disparate methodologies that are hardly comparable and variable results. Another option are dried plasma samples (DPS), which consist of dried plasma collected on filter paper where the only technical requirement is a centrifuge or the use of filter paper that separates blood from plasma such as the Cobas Plasma Separation Card (34, 35).

Another strategy for shortening time-to-diagnosis is point-of-care (POC) assays that can be performed close to or at the site where a patient is receiving healthcare offering fast results, reducing the number of consultations, and the barriers people may face to get tested, increasing linkage to care (36 – 39). The WHO promotes the use of POC HCV RNA assays since they have demonstrated excellent diagnostic performance, promoting access to confirmatory VL testing and treatment (39, 40). Among the POC assays for HCV quantification, the Xpert HCV VL (Cepheid) is very useful in limited infrastructure settings as it requires small equipment and a computer, taking 105 minutes to generate the results from venous plasma (38, 41 – 43). A newer assay (Xpert HCV VL Fingerstick) generates results in <60 minutes from fresh fingerprick capillary blood without the need for venipuncture, facilitating the use at the POC (44, 45). Some studies have evaluated the use of DBS for HCV POC testing (20, 44), but further studies are needed to assess DPS samples and their stability when stored at room temperature.

HCV-Abs detection in DBS/plasma samples has been extensively tested with sensitivity and specificity results close to 95% (21, 46, 47), demonstrating DBS utility in HCV serological diagnosis. However, the evaluation of HCVcAg detection in DBS compared to serum presents discrepant results between studies (10, 11, 48 – 50).

The first aim of this study was to evaluate the performance of DBS and DPS vs plasma for HCV POC RNA detection and quantification using the Xpert HCV VL (Cepheid) and the performance of DBS for HCVcAg detection compared to traditional serum samples using the Architect HCV core antigen assay (Abbott). The second aim was to evaluate the stability of HCV RNA and HCVcAg in DBS and DPS at room temperature and different storage times.

## RESULTS

### Study population

Of the 50 HCV-positive patients (mean age 60.5 years), 42% (21/50) were women and 58% (29/50) were men. Among the 35 subjects with a known country of origin, most came from Spain (83%) and the remaining were from Italy, Ukraine, Venezuela, Morocco, and Romania. Ten patients (20%) were known intravenous drug users. None presented coinfections and two (4%) had cirrhosis. Forty-four patients (88%) had started treatment with either glecaprevir/pibrentasvir or sofosbuvir/velpatasvir 1–3 days prior to the blood sampling. The HCV genotype was known in 28 patients (56%), half of them carrying genotype 1a (50%), 35.7% with 1b, one patient with genotype 2 (3.6%), two patients with genotype 3 (7.1%), and one patient presented mixed infection with genotypes 3–4 (3.6%).

### Detection of HCV RNA in DPS and DBS at different storage times using Xpert HCV VL

**Fig 1:**
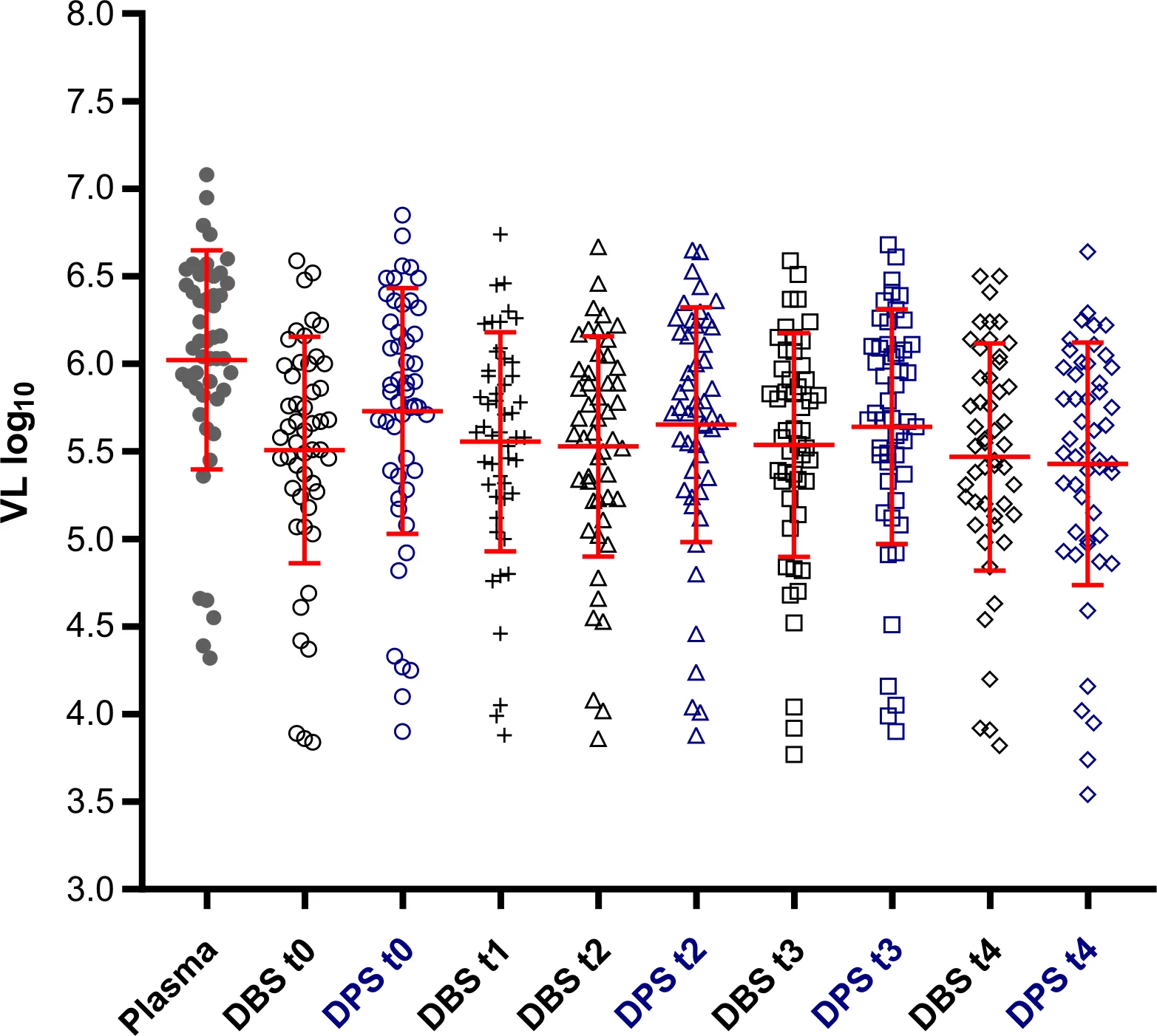
Scatterplots of hepatitis C virus (HCV) RNA levels with Xpert HCV VL in positive plasma samples, dried blood samples (DBS), and dried plasma samples (DPS) at different storage times (*n* = 50): t0 (immediate processing), t1 (7 days of storage), t2 (15 days of storage), t3 (1 months of storage), and t4 (3 months of storage). Data are expressed as mean concentrations with standard deviation ranges. VL, viral load; DPS, dried plasma samples; DBS, dried blood samples.

HCV RNA was detected in all the 50 HCV-positive plasma samples. The VL ranged from 2.09 × 10^4^ IU/mL (log_10_ 4.32) to 1.19 × 10^7^ IU/mL (log_10_ 7.08). All the negative control samples had a VL under the limit of detection (<4 IU/mL). The mean concentration and standard deviation (SD) ranges of HCV RNA levels in the different samples and storage times after volume correction (in DPS) or volume and hematocrit (Hc) correction (in DBS) are shown in Fig. 1.

In the DPS and DBS t0 samples, there were excellent agreement in HCV-RNA detection with 100% sensitivity and 100% specificity compared to plasma as the gold standard sample, even after 3 months of storage at RT. There were no false negative or false positive results, leading to 100% positive predictive value (PPV) and 100% negative predictive value (NPV) (Table S1).

### Differences between HCV VL quantification with Xpert HCV VL in plasma vs DPS and DBS at different storage times

**Fig 2:**
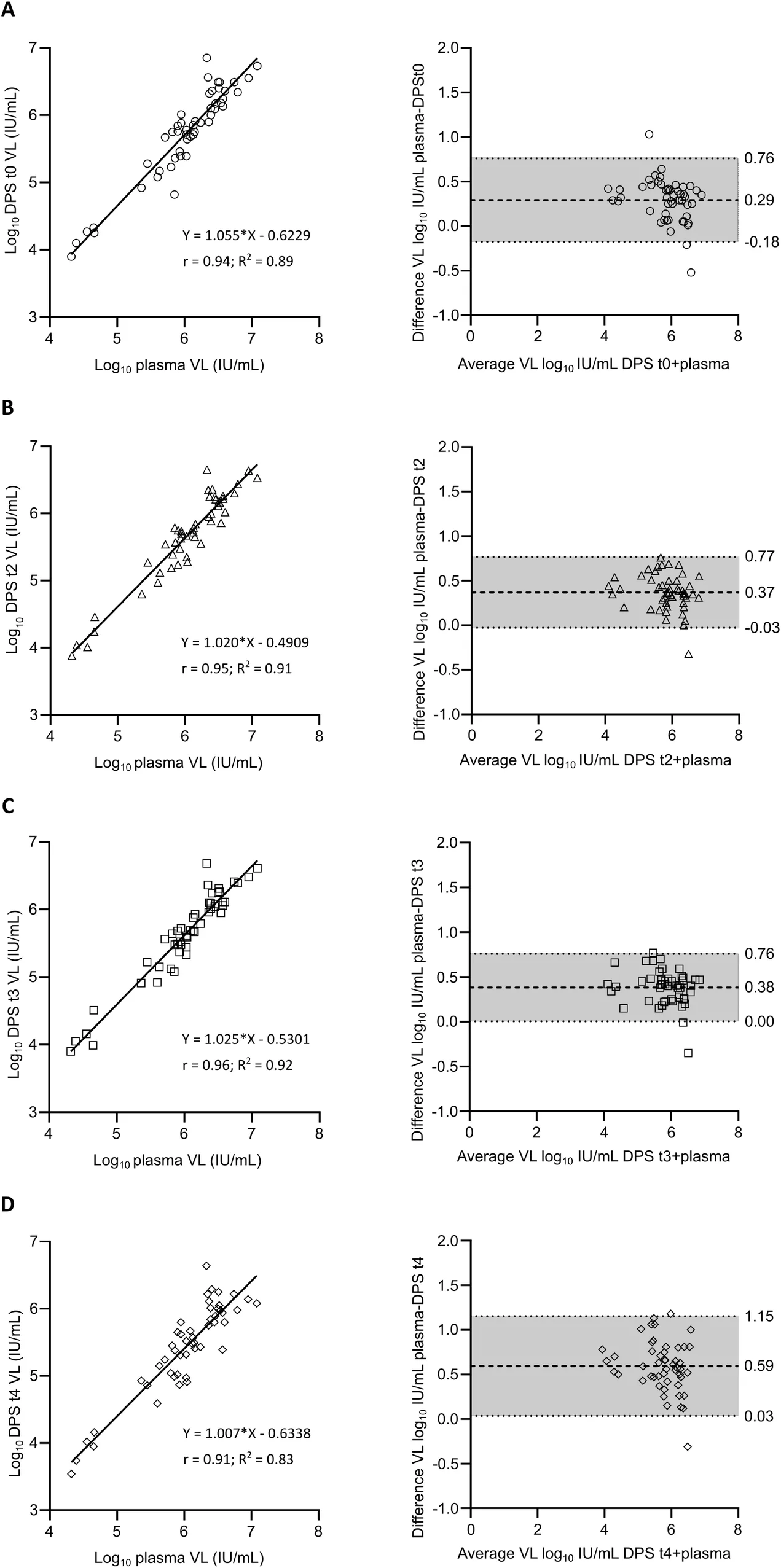
Correlation and Bland-Altman plots between plasma samples and positive dried plasma samples (DPS) at different storage times (*N* = 50). (**A**) DPS t0 (immediate processing). (**B**) DPS t2 (15 days of storage). (**C**) DPS t3 (1 month of storage). and (**D**) DPS t4 (3 months of storage). The mean difference between plasma and DPS is shown in a dashed line. Limits of agreement are shown in the dotted lines. Results obtained with Xpert HCV VL.

There was a good intraclass correlation coefficient between HCV-RNA positive plasma and DPS in all storage times (t0–t4), with a result of 0.86 (*P* < 0.001). Pearson’s correlation of VL results between HCV-RNA positive plasma and DPS varied slightly for the different storage times, showing a statistically significant (*P* < 0.001) strong positive correlation in all of them. The mean VL difference in plasma and DPS was 0.29 log_10_ IU/mL (95% CI: –0.18 to 0.76) at t0 (immediate processing), 0.37 log_10_ IU/mL (95% CI: –0.03 to 0.77) at t2 (15 days of storage), 0.38 log_10_ IU/mL (95% CI: 0.00 to 0.76) at t3 (1 month of storage), and 0.59 log_10_ IU/mL (95% CI: 0.03 to 1.15) at t4 (3 months of storage). Therefore, we observed a slight increase in the VL average difference between samples as the storage time increased, especially at t4 (3 months), but with no false negative results (Fig. 2).

**Fig 3:**
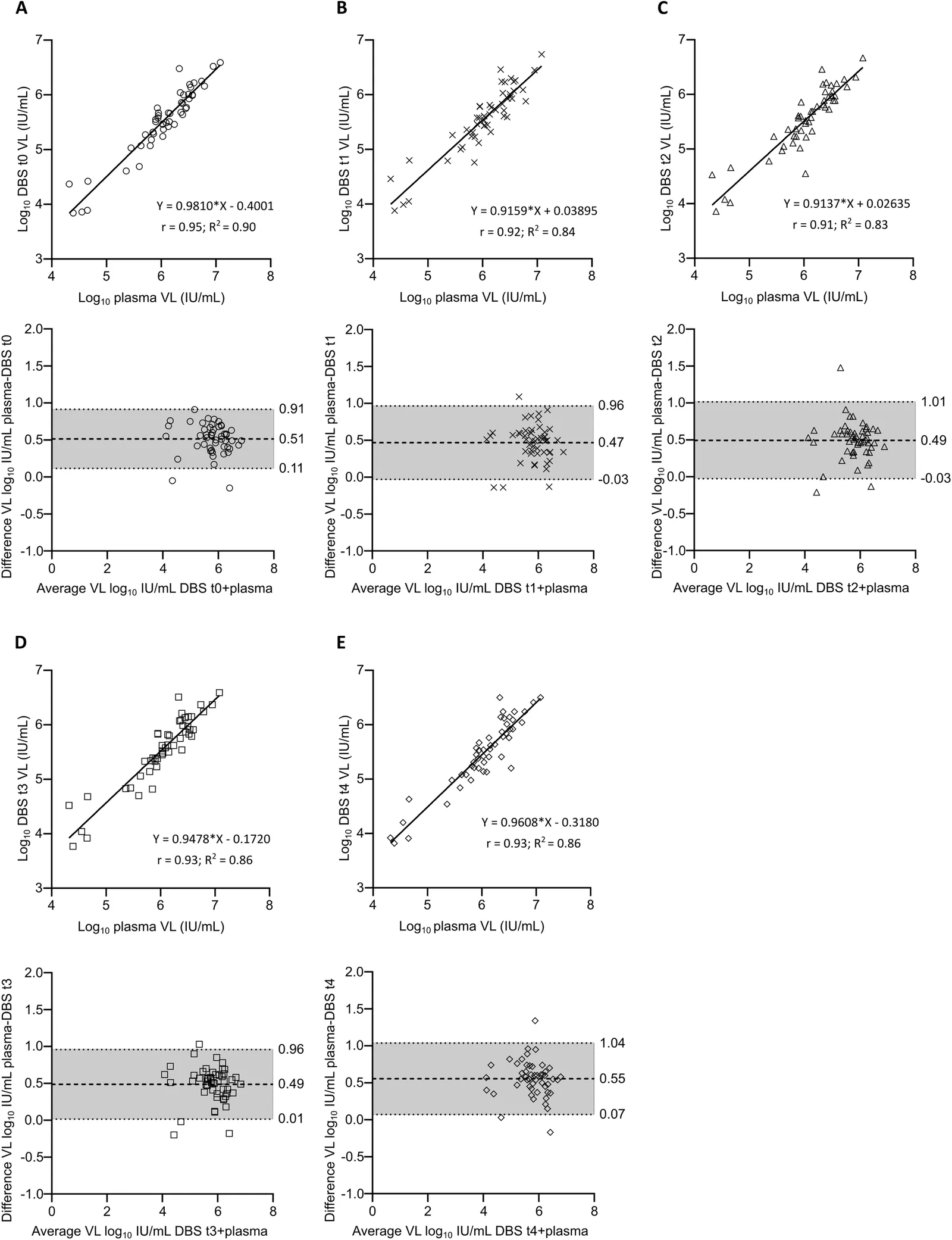
Correlation and Bland-Altman plots between plasma samples and positive dried blood samples (DBS) at different storage times (*N* = 50). (**A**) DBS t0 (immediate processing). (**B**) DBS t1 (7 days of storage). (**C**) DBS t2 (15 days of storage). (**D**) DBS t3 (1 month of storage), and (**E**) DBS t4 (3 months of storage). The mean difference between plasma and DBS is shown in a dashed line. Limits of agreement are shown in the dotted lines. Results obtained with Xpert HCV VL.

We also observed a good intraclass correlation coefficient (ICC = 0.84; *P* < 0.001) between HCV-RNA positive plasma and DBS t0–t4 samples. Pearson’s correlation of VL results between DBS and plasma in HCV-RNA positive samples was slightly lower than for DPS and plasma but still showed a statistically significant (*P* < 0.001) strong positive correlation in all of the storage times. The mean VL difference between plasma and DBS was 0.51 log_10_ IU/mL (95% CI: 0.11 to 0.91) at t0 (immediate processing), 0.47 log_10_ IU/mL (95% CI: −0.03 to 0.96) at t1 (7 days of storage), 0.49 log_10_ IU/mL (95% CI: −0.03 to 1.01) at t2 (15 days of storage), 0.49 log_10_ IU/mL (95% CI: 0.01 to 0.96) at t3 (1 month of storage), and 0.55 log_10_ IU/mL (95% CI: 0.07 to 1.04) at t4 (3 months of storage). Thus, we also found a slight increase in the VL average difference between samples as storage time increased, with no false negative results (Fig. 3).

When comparing HCV VL quantification between plasma samples and DBS (after volume and Hc correction) or DPS (after volume correction), we observed that the average difference in HCV viremia compared to plasma after immediate processing (t0), 15 days (t2), and 1 month (t3) of storage was lower in DPS than in DBS. However, the average difference was more affected by storage time in DPS than in DBS. In addition, after long-term storage (at least 3 months, t4), both DPS and DBS presented a similar reliability for HCV VL quantification, with a slightly lower HCV VL average difference in DBS than in DPS compared to plasma.

### Detection of HCV core antigen in DBS vs serum with Architect HCV Ag assay (Abbott)

**Fig 4:**
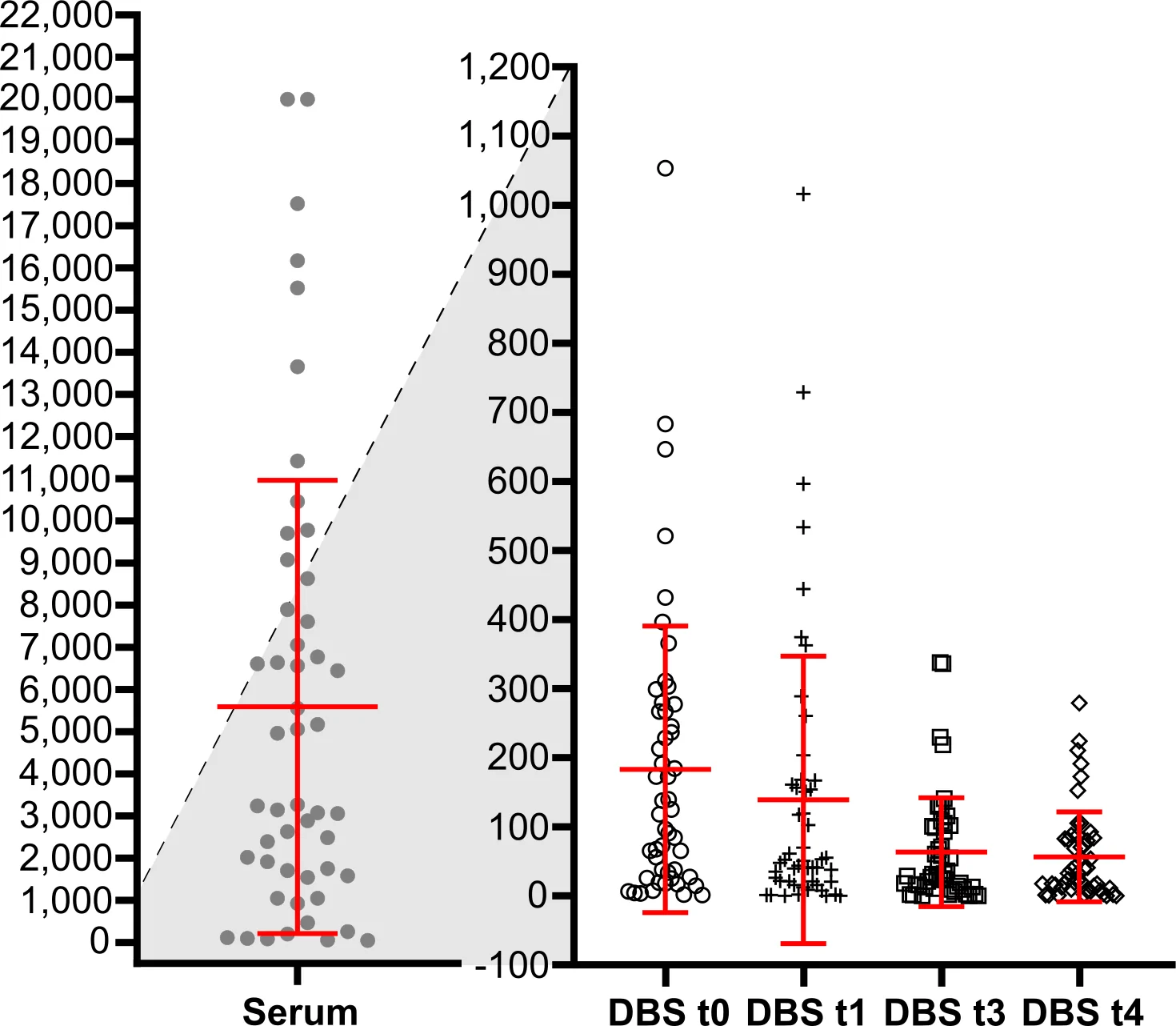
Scatterplots of hepatitis C virus core antigen levels with Architect HCV Ag assay in positive serum samples and dried blood samples (DBS) at different storage times (*n* = 50): t0 (immediate processing), t1 (7 days of storage), t3 (1 month of storage), and t4 (3 months of storage). Data are expressed as mean concentrations with standard deviation ranges. DBS, dried blood samples.

Among the 50 HCV-positive serum samples, the HCVcAg concentration results ranged from 51.78 fmol/L (1.71 log_10_) to 20,000 fmol/L (4.3 log_10_). The mean concentration and standard deviation ranges of HCVcAg in the different samples and storage times are shown in Fig. 4.

In the DBS t0 samples, there were good agreement in HCVcAg detection with 96% sensitivity and 100% specificity compared to serum as the gold standard. There were two false negative results and no false positive results, leading to a 100% positive predictive value (PPV) and 91% negative predictive value (NPV) (Table 1).

**TABLE 1:** Hepatitis C virus core antigen detection in positive and negative DBS after immediate processing (t0) vs serum as gold standard^*a*^

		HCVcAg detection in DBS t0 (*n* = 70)	
HCVcAg detection in serum (*n* = 70)		Positive	Negative
Positive	50	48	2
Negative	20	0	20

^*a*^ HCVcAg detection by the Architect HCV Ag assay (Abbott). Positive cut-off value 3 fmol/L. HCV, hepatitis C Virus; DBS, dried blood sample; HCVcAg, HCV core antigen.

The two false negative results at t0 corresponded to samples from two HCV-infected subjects under treatment with glecaprevir/pibrentasvir with an HCVcAg serum concentration of 57.74 fmol/L (VL 4.55 Log IU/mL and HCV genotype 3–4 coinfection) and 6635.77 fmol/L (VL 5.82 Log IU/mL, unknown genotype), respectively.

At the following storage times, the sensitivity of DBS decreased to 86% (t1, seven false negative results), 84% (t3, eight false negative results), and 86% (t4, seven false negative results), as shown in Table 2.

**TABLE 2:** Sensitivity of hepatitis C virus core antigen detection in DBS at different storage times vs serum as gold standard^*a*^

	HCVcAg detection in positive samples (Positive cut-off 3 fmol/L)
**Time of storage**	**DBS Sensitivity (95% CI)**
t0 (0 d)	96% (86%-99%)
t1 (7 d)	86% (73%-94%)
t3 (1 mo)	84% (71%-93%)
t4 (3 mo)	86% (73%-94%)

^*a*^ HCVcAg detection by the Architect HCV Ag assay (Abbott). HCV, hepatitis C virus; HCVcAg, hepatitis C virus core antigen. DBS, dried blood samples. CI, confidence intervals.

### Correlation between HCV core antigen in serum and HCV RNA VL in plasma

**Fig 5:**
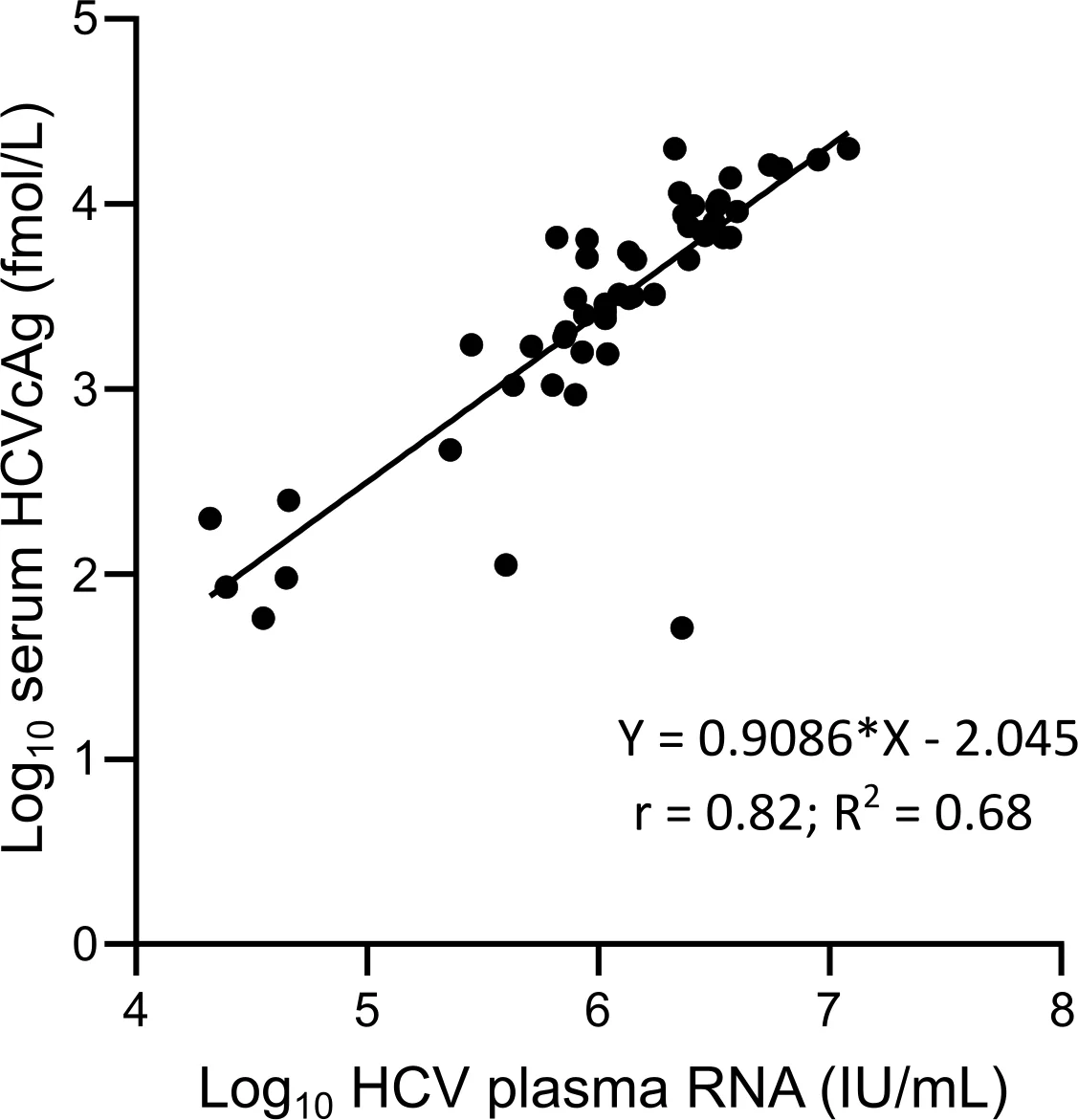
Pearson’s correlation between hepatitis C virus (HCV) core antigen (HCVcAg) in serum and HCV RNA in plasma. Hepatitis C virus core antigen (HCVcAg) tested with Architect HCV Ag assay. HCV RNA viral load tested with Xpert HCV VL.

The correlation between the HCVcAg in serum and HCV RNA in plasma in the HCV-RNA positive samples was moderate and statistically significant (*P* < 0.001) (Fig. 5).

### Correlation between HCV core antigen and HCV RNA VL in DBS at different storage times

We observed a strong to moderate positive correlation between HCV RNA detected with Xpert HCV VL and HCVcAg detected with Architect HCV Ag assay (Abbott) in DBS for the different storage times: in t0 (*r* = 0.90, *R^2^* = 0.80, Y = 0.005538X + 36.64), t1 (*r* = 0.90, *R^2^* = 0.81, Y = 0.004898X − 2.401), t3 (*r* = 0.82, *R^2^* = 0.68, Y = 0.001971X + 9.022), and t4 (*r* = 0.81, *R^2^* = 0.66, Y = 0.001738X + 13.53), being *P* statistically significant (*P* < 0.001) in all these storage times (Fig. S1).

**Fig 6:**
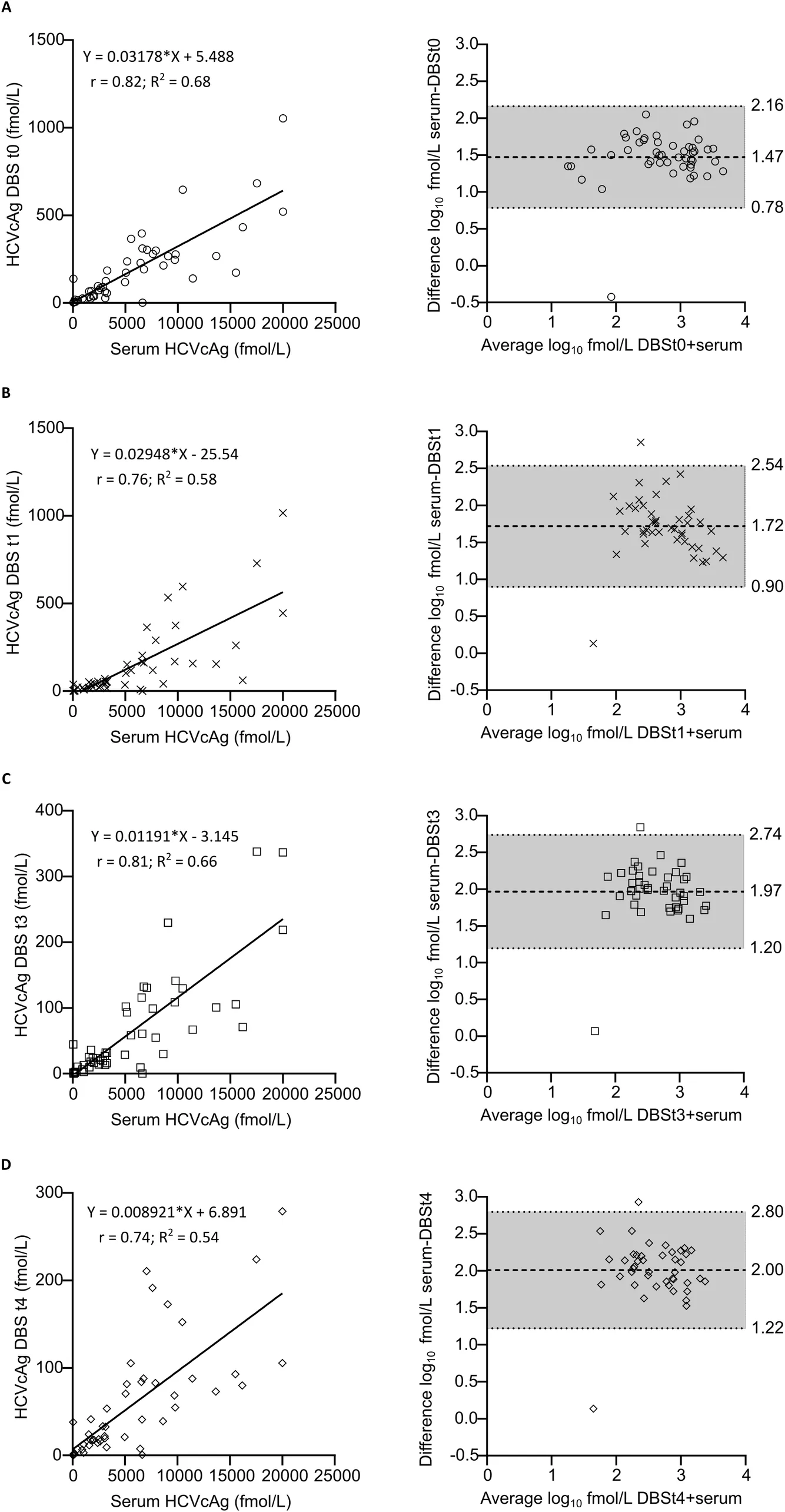
Correlation and Bland-Altman plots between positive serum samples and dried blood samples (DBS) at different storage times. (**A**) DBS t0 (immediate processing). (**B**) DBS t1 (7 days of storage). (**C**) DBS t3 (1 month of storage). (**D**) DBS t4 (3 months of storage). Pearson’s correlation was performed in all serum positive samples (*N* = 50). Bland-Altman plots were performed with DBS positive samples: (**A**) *N* = 48, (**B**) *N* = 43, (**C**) *N* = 42, and (**D**) *N* = 43. The mean difference between serum and DBS is shown in a dashed line. The limits of agreement are shown in the dotted lines. HCVcAg, Hepatitis C virus core antigen. Hepatitis C virus core antigen (HCVcAg) tested with Architect HCV Ag assay.

The HCVcAg results between DBS and serum in HCV-positive samples showed a positive moderate correlation in t0 and t3 and a weaker correlation in t1 and t4 (*P* < 0.001 in all the storage times). The mean HCVcAg difference between serum and DBS at the different storage times was 1.47 log_10_ fmol/mL ± 0.35 (95% CI: 0.78 to 2.16) in t0 (immediate processing), 1.72 log_10_ fmol/mL ± 0.42 (95% CI: 0.90 to 2.54) in t1 (7 days of storage), 1.97 log_10_ fmol/mL ± 0.39 (95% CI: 1.2 to 2.74) in t3 ± 0.24 (1 month of storage), and 2.01 log_10_ fmol/mL ± 0.40 (95% CI: 1.22 to 2.80) in t4 (3 months of storage), showing an increase in the HCVcAg average difference between samples as storage time increased (Fig. 6).

### Identification of an optimized cut-off value for Architect HCV Ag assay to improve the sensitivity

**Fig 7:**
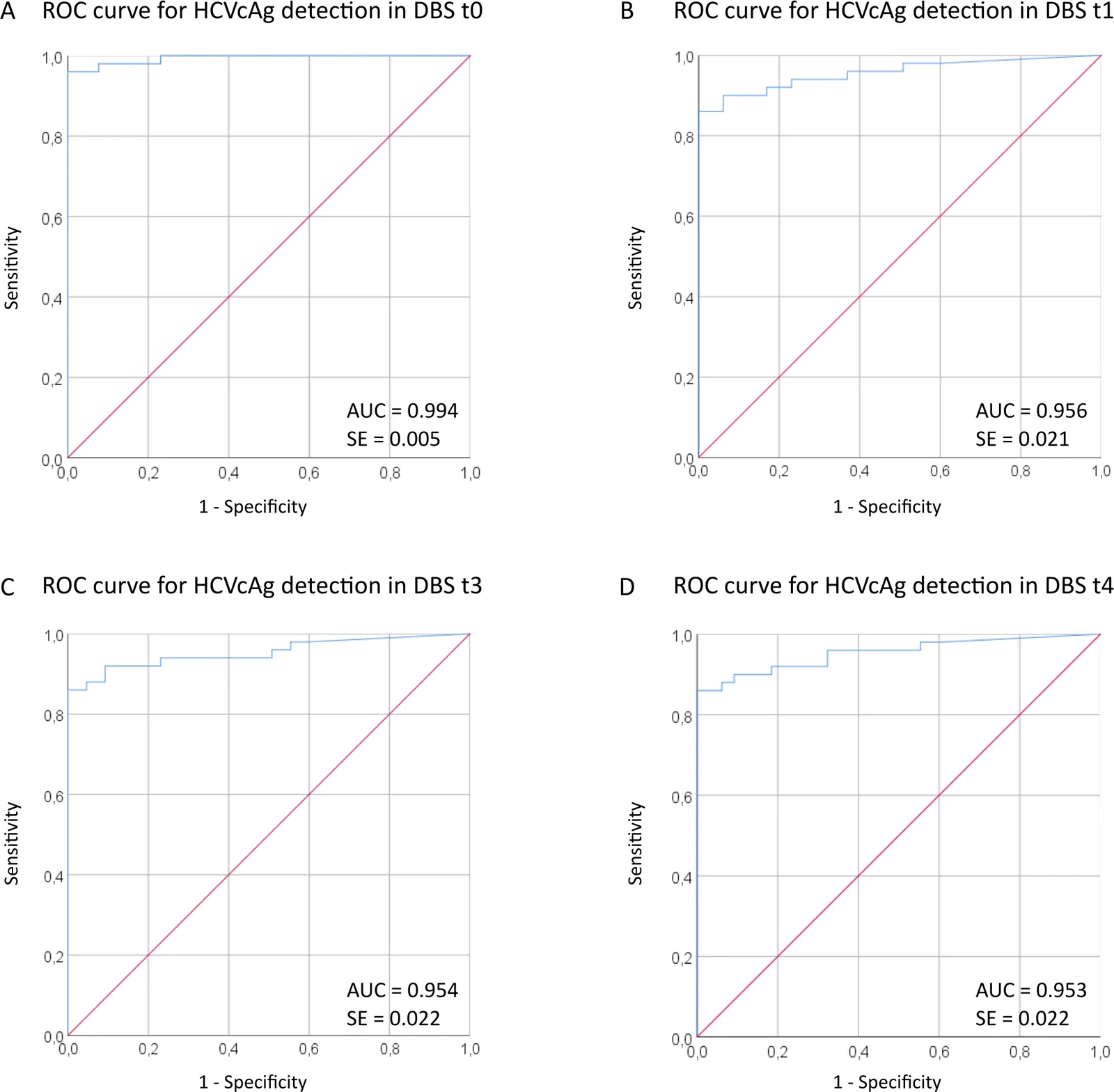
Receiver operating characteristic (ROC) curves for hepatitis C virus core antigen detection in DBS with Architect HCV Ag assay and different storage times at room temperature. The area under the curve and standard error are indicated for each curve. AUC, area under the curve; SE, standard error; t0, immediate processing; t1, 7 days of storage; t3, 1 month of storage; t4, 3 months of storage; HCVcAg, hepatitis C virus core antigen.

Receiver operating characteristic (ROC) curves were calculated for each storage time. Results showed an area under the curve (AUC) of 0.99 in t0 and above 0.95 in the rest of the studied storage times. As described in the Materials and Methods section, the same 65 negative controls were used to calculate the ROC curves for every storage time as false positive results were not expected to arise after storage (Fig. 7).

Since the sensitivity of HCVcAg detection in DBS decreased to 86% after 7 days to 3 months of DBS storage at room temperature (Table 3), we wanted to explore alternative cut-offs to increase the Architect HCV Ag assay’s sensitivity for HCVcAg screening in stored DBS. For this purpose, we tested different cut-offs according to the ROC curves. As there were no false positive results in the 65 negative controls tested, the maximum specificity was obtained using the standard cut-off. Modifying the Architect cut-off values we obtained a sensitivity ≥90% and a PPV ≥81% for HCVcAg detection in DBS stored for up to 3 months at RT until testing. The sensitivity, specificity, positive predictive value, and negative predictive value of the standard cut-off according to Architect, the maximum sensitivity cut-off, and our optimized cut-off are described in Table 3 for each DBS storage time.

**TABLE 3:** Standard, maximum sensitivity, and recommended cut-offs for HCVcAg detection in DBS after different storage times at room temperature^*a*^

Storage time	Cut-off	Value (fmol/L)	Sen	Spe	PPV	NPV
t0 (immediate processing)	Standard	3	96%	100%	100%	97%
	Max sensitivity	1.22	100%	77%	77%	100%
	Recommended	1.935	98%	92%	91%	98%
t1 (7 days of storage)	Standard	3	86%	100%	100%	90%
	Max sensitivity	0.19	98%	49%	60%	97%
	Recommended	1.52	92%	83%	81%	93%
t3 (1 mo of storage)	Standard	3	84%	100%	100%	89%
	Max sensitivity	0.06	98%	45%	58%	97%
	Recommended	1.755	92%	91%	88%	94%
t4 (3 mo of storage)	Standard	3	86%	100%	100%	90%
	Max sensitivity	0.055	98%	45%	58%	97%
	Recommended	1.815	90%	91%	88%	92%

^*a*^ HCVcAg detection by the Architect HCV Ag assay (Abbott). Serum values as the gold standard. Sen, sensitivity; Spe, specificity; PPV, positive predictive value; NPV, negative predictive value; HCVcAg, hepatitis C virus core antigen.

## DISCUSSION

Dried blood collected on filter paper is a useful alternative sample to whole blood or plasma for HCV diagnosis and monitoring in settings lacking adequate laboratory infrastructure (24) or in high-income settings with high-risk groups for HCV infection with limited access to medical care, such as undocumented immigrants, homeless people, people in prisons, sex workers, and people who inject drugs, among others.

Molecular POC tests for HCV RNA detection present excellent accuracy for hepatitis diagnosis (38, 51). Previous studies have shown that HCV RNA can be reliably detected and quantified with the molecular POC Xpert HCV VL test in DBS with sensitivities around 90% (38, 42, 52). In this study, we found maximum (100%) sensitivity and specificity by Xpert HCV VL in DBS and DPS after immediate processing (t0) even before volume (DPS) or hematocrit (Hc) and volume (DPS) corrections, demonstrating excellent reliability in both samples for HCV POC RNA detection.

Sometimes dried samples cannot be processed immediately after sampling. More studies on the performance of DBS conducted in different settings and with varying storage conditions are still needed (23). For this reason, we tested the samples after different storage times at room temperature. As for the filter paper used for sampling DBS, 903 Protein Saver cards have been reported as a cheap and useful support for dried blood samples and they can last up to 9 months at room temperature (53). The impact of storage time at RT for HCV VL quantification varies across studies. Some authors found little variation between HCV RNA ct values in VL quantification irrespective of storage temperature or length of storage (54), suggesting a high HCV RNA stability in DBS after prolonged periods (up to 1 year) of storage at room temperature. However, other authors reported HCV VL decrease in DBS after storage time. One study observed a reduction of Xpert HCV VL by 0.29 ± 0.12 log IU/mL after the DBS storage at room temperature for 7 days before freezing (52). Another study observed an average HCV RNA concentration loss of 4.4 log_10_ IU/mL between stored DBS and plasma samples after 60 days of storage under different temperature conditions, recommending using freshly prepared DBS for RNA detection (55).

In this study, we found a 100% sensitivity for HCV POC RNA detection in DBS and DPS in all the tested storage times, proving an excellent sensitivity even after 3 months of storage at room temperature. The sensitivity and specificity in DBS and DPS for t0 were also 100% before volume/Hc correction. However, volume correction in DPS and volume and Hc correction in DBS convert HCV RNA IU per DBS dot to HCV RNA IU per milliliter of plasma, which are the same units as plasmatic viremia, allowing to compare plasma and dried samples RNA quantification with greater accuracy. Thus, we strongly recommend performing both corrections when using dried samples to quantify HCV VL.

Even after volume (DPS)-Hc/volume (DBS) correction, VL values in dried samples vary when compared to plasma, as previously reported (52, 54). In our study, compared to plasmatic VL, corrected HCV VL in DPS was more accurate than DBS after immediate processing (t0) (VL average difference of 0.3 log_10_ IU/mL vs 0.5 log_10_ IU/mL). In the following storage times, the mean of the VL average differences compared to plasma was similar in both dried samples (0.4 log_10_ IU/mL in DPS vs 0.5 log_10_ IU/mL in DBS) with an increase in the VL average difference in longer storage times. However, in DPS, there was a significant rise in the VL average difference for t4 (3 months of storage) and when this time was excluded, the mean of the VL average differences compared to plasma was reduced to 0.3 log_10_ IU/mL. DBS VL variation between storage times was smaller, with a VL decrease not greater than 0.05 log IU/mL, suggesting that after the initial reduction in viral load, DBS are more stable over time than DPS.

In some settings, PCR techniques may not be available or be too expensive, and HCV detection must be carried out with serological techniques such as antibody and HCVcAg detection. HCV Ab detection in DBS has shown excellent sensitivity (above 98%) in previous studies even when stored at room temperature, being greater than HCVcAg detection in DBS (around 6% less sensitivity) (12). Nevertheless, HCVcAg has two main advantages over Ab detection: it can tell active infection as it’s a serological indicator of active replication and it can be detected within 15 d after infection, shortening the window period by 5–7 weeks compared to HCV Ab detection (56, 57). Furthermore, HCVcAg detection is easy to operate, time-saving, and low-cost compared with HCV RNA detection, with similar clinical sensitivity (56).

Many studies correlate HCVcAg levels to HCV RNA VL (58 – 62), with moderate to strong positive correlations between them. In our study, there was a moderately strong correlation between HCV RNA VL in plasma and HCVcAg concentration in serum (*r* = 0.82, *P* < 0.001). We also observed a strong correlation between RNA VL and HCVcAg in DBS that were immediately processed and stored at room temperature for 7 days, being this correlation weaker for samples stored for 1 and 3 months.

As for HCV core antigen detection results, the concentration values decreased greatly in DBS compared to serum samples (1.5 log_10_ fmol/L in t0 up to two log_10_ fmol/L in t4). However, the detection of HCVcAg in DBS by the Architect HCV Ag assay was good in t0 with 96% sensitivity and 100% specificity, demonstrating that HCVcAg detection in DBS can be useful as a qualitative HCV detection test. A similar study comparing Architect’s HCVcAg detection in plasma and DBS (with no further storage times) in Australia showed 88.6% sensitivity and 97% specificity, observing higher HCVcAg levels in plasma than in DBS (49). Another study performed in Tanzania showed lower sensitivity (76.1%) and specificity (97.3%) with an AUC of 0.87 (10), whereas in a Vietnamese study, sensitivity and specificity of HCVcAg detection in DBS were 87% and 100%, respectively (50). However, the high temperatures and humidity in these areas may have a detrimental effect on HCVcAg stability.

In the following storage times, the HCVcAg sensitivity in DBS decreased by around 10% using the standard cut-off value (Table 2). Using lower cut-offs, as the recommended cut-off described in Table 3, the sensitivity could be increased above 90% while keeping specificity values ranging from 83% to 92%, allowing the use of DBS for HCVcAg detection as a good screening method when PCR-based POC techniques or serum/plasma samples are not available. Regardless of the time of storage when using the recommended cut-offs for HCV screening, the positive samples with a value between the recommended cut-off and the standard cut-off (3 fmol/L) should be confirmed with a second technique. Manufacturers’ instructions recommend retesting or confirming HCVcAg values between 3 and 10 fmol/L when using traditional samples. In our study, we observed no DBS false positive results above 3 fmol/L. As the DBS format may reduce the sensitivity but not the specificity when detecting HCVcAg, patients with an HCVcAg concentration value above 3 fmol/L should be considered positive for HCV.

One limitation of this study was that we did not have the genotype data of most of the positive samples and therefore could not assess the effect of HCV genotype on the results of the Architect assay. Since the target population for the HCV testing using DBS or DPS are treatment-naïve patients with HCV infection and these usually present high viral loads, our HCV-RNA positive patient cohort with high HCV VL (ranging from 4 to 7 log) is a realistic representation of a first diagnosis situation. However, in some cases, naïve patients can present low HCV VL, which has been associated with better outcomes (63). To study the performance of dried samples in these cases and certain HCV chronic infections, new study cohorts, including patients with HCV viremia and antigenemia close to the assays’ LOD (4 UI/mL for Xpert HCV VL and 3 fmol/L for Architect) should be analyzed in the future, allowing to explore the impact on the assay lower limit of detection (LOD) of the low specimen volume present in dried samples.

In conclusion, HCV RNA detection by the PCR-POC test Xpert HCV VL assay in DBS and DPS was a highly sensitive and specific technique for HCV diagnosis even after 3 months at room temperature and can be very useful in settings with decentralized diagnosis or vulnerable populations with difficult access to healthcare. For VL quantification after volume and hematocrit correction, the reduction of VL compared to plasma was lower in DPS than in DBS. Thus, we recommend collecting DPS in case of having a centrifuge and venipuncture capacity or plasma separation cards using capillary fingerprick blood, provided that it is not going to be stored for more than 1 month. However, DBS seem more stable after RT storage and would be the appropriate sample for long-term storage (at least for 3 months). For monitoring purposes, we recommend using the same sample format for the patient’s follow-up. As for the DBS use for HCVcAg detection, they can be useful for qualitative HCV diagnosis even after RT storage. Although HCVcAg detection in DBS has a reduced sensitivity compared to DBS and DPS RNA detection, its sensitivity and specificity are good and sensitivity can be further increased when optimizing the standard cut-off, being an interesting alternative for HCV screening in resource-limited settings with no PCR availability. Despite HCVcAg showing a good correlation with HCV RNA in plasma and serum samples, we recommend caution when trying to infer VL from HCVcAg concentrations in DBS, discouraging this practice for DBS stored longer than 1 week. Furthermore, DBS can be easily incorporated into reflex-testing and pooling strategies for population HCV screening (64, 65), being a useful tool in the roadmap to accomplish the WHO proposed HCV elimination by 2030.

Finally, the main aim of an alternative HCV sample format is to reach communities with difficult or limited access to healthcare and provide a quick diagnosis to start treatment as soon as possible and avoid patient loss. Since sample collection and transport to the testing facility should be as fast as possible, and based on our findings, we recommend not to exceed 1 month of room temperature storage of DBS or DPS for HCV VL quantification, reducing this time to 7 days for DBS if HCVcAg detection is going to be performed. Longer term DBS/DPS storage, even when feasible, should be limited to research purposes or further testing of other diseases when not available on-spot.

## MATERIALS AND METHODS

This prospective case-control study was carried out at the Ramón y Cajal University Hospital (Madrid, Spain), a public hospital with a high number of HCV-infected patients under treatment (206 treated between March 2019 and January 2020). For this study, 70 patients were consecutively enrolled during 1 year (June 2021 to June 2022) at the gastroenterology consultation upon signing the informed consent, 50 HCV-positive and 20 HCV-negative (controls). Positive HCV patients had been previously diagnosed in the Microbiology Department using Alinity_s Anti-HCV II assay to detect HCV antibodies, performed on the Abbott Alinity_s platform (Abbott Diagnostics) for HCV screening, followed by HCV quantitative detection of HCV core antigen (performed with Architect HCV core Ag assay, Abbott Diagnostics) in those with positive serology to detect active HCV infection. If the HCV core Ag assay was not conclusive (gray zone), then molecular HCV detection was performed in Roche 6800 platform (Roche Diagnostics), following the manufacturer’s recommendations. The sample size was calculated according to the available budget for the project and the number of positive patients that we were able to recruit in the established time for the project. HCV-negative patients were both anti-HCV antibody-negative and HCV RNA-negative.

Forty-five additional negative controls with available DBS were added for the Architect HCVcAg testing to establish an adjusted cut-off for screening purposes. Ethylenediaminetetraacetic acid (EDTA) anticoagulated venous blood, serum, and plasma samples were drawn after obtaining the signed informed consent of the patient to participate in the study. The study protocol was conducted under the Declaration of Helsinki and Good Clinical Practice guidelines and was approved on 4 May 2020 by the Hospital’s Medical Research Ethics Committee (CEIm 2014/0078).

**Fig 8:**
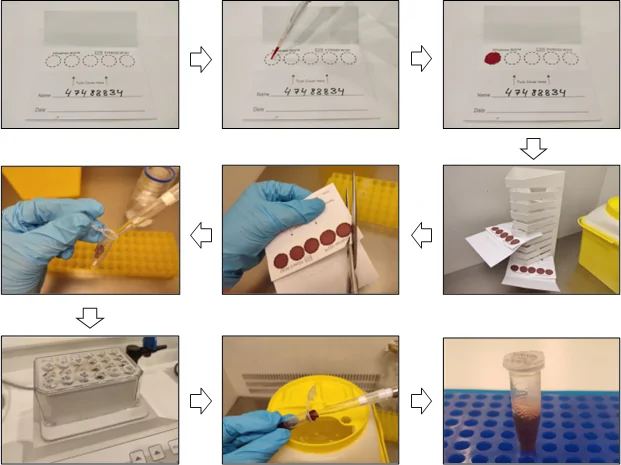
Processing of dried blood samples. Each dot was spotted on the 903 filter paper cards with 70 µL of EDTA venous blood. Cards were dried overnight at room temperature and stored until processing. One dot was removed and inserted in a microtube with 1 mL of GeneXpert lysis buffer for viral load testing (2 dots and 500 µL of phosphate-buffered saline were used for HCVcAg testing). Microtubes were incubated for 1 hour with gentle rotation at room temperature. The paper disks were removed before performing the corresponding test on the elution.

As illustrated in Fig. 8, DBS were prepared by spotting 70 µL of EDTA venous blood in each dot on 903 filter paper cards (Schleicher & Schuell BioScience GmbH, Barcelona, Spain). DPS were prepared by spotting 50 µL of plasma in each dot on the same filter paper cards. Cards were dried overnight at room temperature and stored in individual plastic bags with desiccant bags at −80°C until processing (for a maximum of 3 months). A card was prepared for the 50 HCV-positive patients for each different room temperature storage time: immediate processing (t0), 7 days (t1), 15 days (t2), 1 month (t3), and 3 mo (t4) (Table 4).

**TABLE 4:** Room temperature storage times and samples analyzed for RNA and antigen detection^*a*^

	Storage times and samples				
	t0	t1	t2	t3	t4
Analysis performed	0 days	7 days	15 days	1 mo	3 mo
HCV core antigen Architect HCV Ag assay (Abbott)	50 HCV+ serum 20 HCV- serum 50 HCV+ DBS 65 HCV- DBS	50 HCV+ DBS		50 HCV+ DBS	50 HCV+ DBS
HCV viral load Xpert HCV VL (Cepheid)	50 HCV+ plasma 20 HCV- plasma 50 HCV+ DBS 20 HCV- DBS 50 HCV+ DPS 20 HCV- DPS	50 HCV+ DBS	50 HCV+ DBS 50 HCV+ DPS	50 HCV+ DBS 50 HCV+ DPS	50 HCV+ DBS 50 HCV+ DPS

^*a*^ HCV, hepatitis C virus; DBS, dried blood samples; DPS, dried plasma samples.

For VL testing, one DPS dot and one DBS dot were removed from the card for each patient and both inserted in two different microtubes with 1 mL of GeneXpert lysis buffer each. For HCVcAg testing, two DBS dots were removed from the card for each patient and inserted in a microtube with 500 µL of phosphate-buffered saline (PBS). For both tests, microtubes were incubated for 1 hour with gentle rotation at room temperature. After removing the paper disks, the elution was processed according to the manufacturer’s instructions.

Plasma, DBS, and DPS were processed for HCV RNA detection and quantification using the POC quantitative assay Xpert HCV Viral Load Assay (Xpert HCV VL, Cepheid) according to the manufacturer’s instructions. This test has a lower limit of detection of HCV RNA of 4.0 IU/mL in EDTA plasma and 6.1 IU/mL in serum and presents a limit of quantitation (LOQ) of 10 IU/mL, and 100% sensitivity and 97% specificity for HCV RNA detection in plasma. It is able to quantify HCV genotypes 1–6 over the range of 10 to 100,000,000 IU/mL (39). To test DBS and DPS performance at different storage times, the paper cards were stored at room temperature (approximately 25°C) for 0 day (t0), 15 days (t2), 1 month (t3), and 3 months (t4). An additional storage time was included for DBS cards, storing them for 7 days (t1) (Table 4). We decided not to include t1 (7 days) in DPS since previous studies in HIV supported that RNA was stable in DPS for RNA testing during 1 week without cold storage (28, 66).

DBS and DPS negative controls for virological testing were tested only for t0 storage time. When the room temperature storage time was completed, all samples were stored at −80°C until diagnostic testing. Viremia results were log-transformed before statistical analysis. Volume correction was performed to quantify HCV VL in DPS and DBS, considering also the Hc in DBS using the following formula: (result × sample volume)/(dot volume × (100–Hc)/100). In this way, we converted the HCV IU per DBS dot into HCV IU per plasma milliliter, obtaining the VL present in the plasma fraction contained in the 70 µL blood per dot as previously reported (67, 68).

Serum and DBS were processed for HCVcAg determination using the Architect i2000SR and Architect HCV Ag assay (Abbott Laboratories, Germany) with an LOD of 3 fmol/L, a specificity of ≥99.5%, and a sensitivity of 97.8% in serum and plasma according to the manufacturer’s instructions. DBS were stored at room temperature for 0 day (t0), 7 days (t1), 1 month (t3), and 3 months (t4) (Table 4). DBS HCVcAg negative controls were tested only for t0 storage time. When the room temperature storage time was complete, all samples were stored at −80°C until diagnostic testing. Qualitative results for both serum and DBS were interpreted according to the Architect HCV Ag assay manufacturer’s cut-off value (S/CO value of ≥3.00 fmol/L). Results between 3 and 10 fmol/L (gray-zone) were retested according to manufacturer’s instructions, if the two results of the sample were ≥3.00 fmol/L, the sample was considered positive. No volume correction was applied for HCVcAg detection.

We performed statistical analysis using IBM SPSS Statistics v.28.0.1.1 and GraphPad v.8. Sensitivity and specificity parameters of the RNA assay in DBS and DPS were estimated using the results of the plasma samples as the gold standard. The sensitivity and specificity parameters of the HCVcAg determination were estimated using the serum samples as the gold standard. Bland-Altman and Intraclass Correlation Coefficient with one-way random effects model analysis were performed to compare VL in DBS/DPS vs plasma and HCVcAg results in DBS vs serum after log transformation. ROC curves and AUC were calculated for each storage time using a total of 65 negative controls. The correlation coefficients between HCV RNA and HCVcAg were calculated by Pearson’s test. A *P* value of < 0.05 was considered statistically significant.

## SUPPLEMENTAL MATERIAL

Figure S1 spectrum.01748-23-s0001.pdf Pearson correlation between hepatitis C virus (HCV) core antigen (HCVcAg) and HCV RNA in DBS after different storage times.

Table S1 spectrum.01748-23-s0002.pdf Hepatitis C Virus RNA detection with Xpert HCV VL in HCV-positive and HCV-negative DPS and DBS.

ASM does not own the copyrights to Supplemental Material that may be linked to, or accessed through, an article. The authors have granted ASM a non-exclusive, world-wide license to publish the Supplemental Material files. Please contact the corresponding author directly for reuse.
