# Supplementary material for: Impact of storage time in dried blood samples (DBS) and dried plasma samples (DPS) for point-of-care hepatitis C virus (HCV) RNA quantification and HCV core antigen detection
Source: Microbiol Spectr. 2023 Sep 1;11(5):e01748-23. doi: 10.1128/spectrum.01748-23 (PMC10581200; doi:10.1128/spectrum.01748-23)
Supplement: Figure S1 — Pearson correlation between hepatitis C virus (HCV) core antigen (HCVcAg) and HCV RNA in DBS after different storage times. [file spectrum.01748-23-s0001.pdf]

Figure S1. **Pearson correlation between hepatitis C virus (HCV) core antigen (HCVcAg) and HCV RNA in DBS after different storage times**

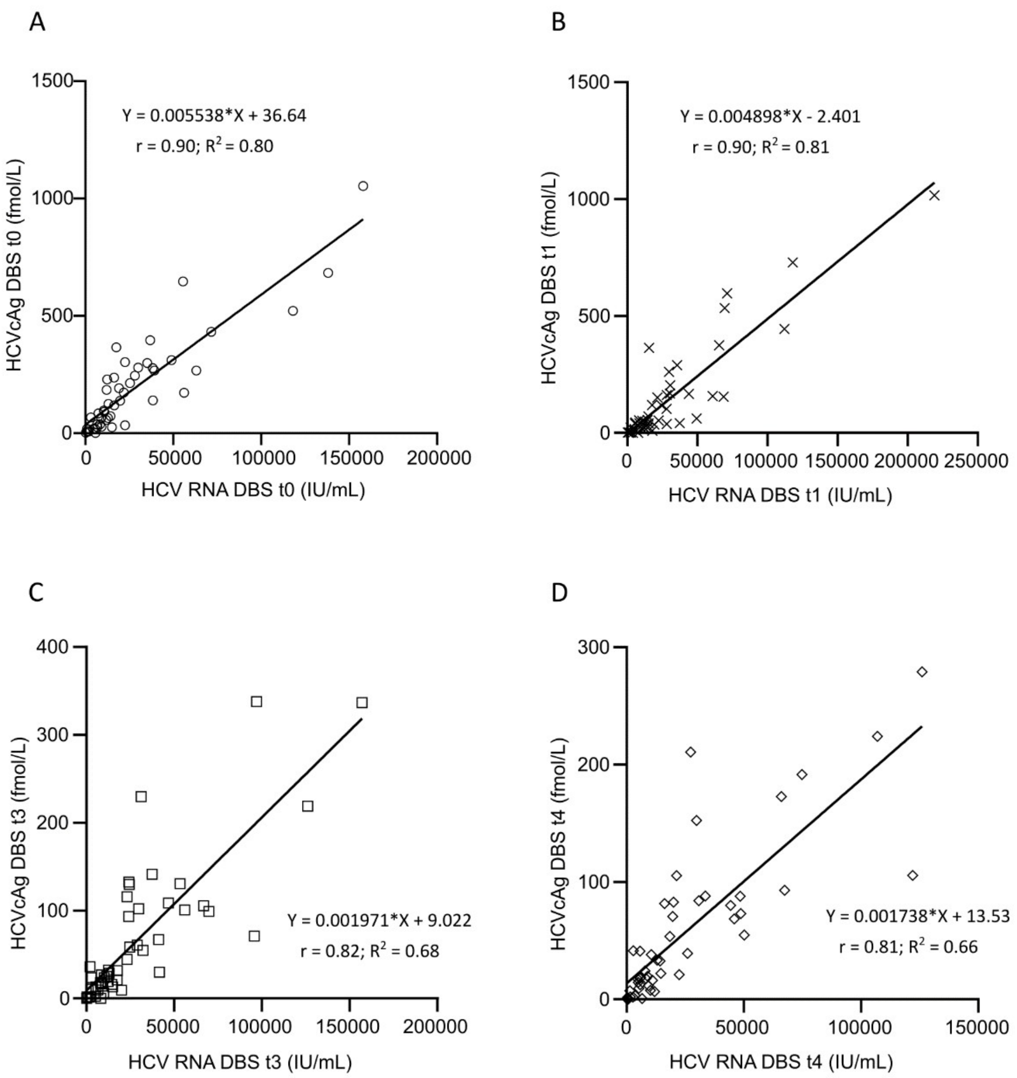

Figure S1 legend: (A) Immediate processing or t0 (B) 7 days of storage or t1 (C) 1 month of storage or t3 (D) 3 months of storage or t4. Hepatitis C virus core antigen (HCVcAg) tested with Architect HCV Ag assay. HCV RNA viral load tested with Xpert HCV VL.
