# Supplementary material for: Impact of storage time in dried blood samples (DBS) and dried plasma samples (DPS) for point-of-care hepatitis C virus (HCV) RNA quantification and HCV core antigen detection
Source: Microbiol Spectr. 2023 Sep 1;11(5):e01748-23. doi: 10.1128/spectrum.01748-23 (PMC10581200; doi:10.1128/spectrum.01748-23)
Supplement: Table S1 — Hepatitis C Virus RNA detection with Xpert HCV VL in HCV-positive and HCV-negative DPS and DBS. [file spectrum.01748-23-s0002.pdf]

Table S1. Hepatitis C Virus RNA detection with Xpert HCV VL in HCV-positive and HCV-negative DPS and DBS on t0 (immediate processing)[a]

		**HCV RNA detection in DPS t0** (n=70)		**HCV RNA detection in DBS t0** (n=70)	
**HCV RNA detection in plasma** (n=70)		Positive	Negative	Positive	Negative
Positive	50	50	0	50	0
Negative	20	0	20	0	20

[a]Xpert HCV VL limit of detection: 4 IU/mL, limit of quantitation: 10 IU/mL. Gold standard: plasma samples. HCV, Hepatitis C Virus; DPS, dried plasma samples; DBS, dried blood samples.
